# Preoperative bilateral drainage for perihilar cholangiocarcinoma via endoscopic ultrasound-guided hepaticogastrostomy using a novel side-hole plastic stent

**DOI:** 10.1055/a-2794-0225

**Published:** 2026-02-26

**Authors:** Takuya Doi, Hirotoshi Ishiwatari, Akihiro Ohba, Hiroki Sakamoto, Masahiro Yamamura, Teichi Sugiura

**Affiliations:** 1 38471 Division of Pancreatobiliary Medicine, Shizuoka Cancer Center, Shizuoka, Japan; 2 Division of Hepato-Pancreatic Surgery, Shizuoka Cancer Center, Shizuoka, Japan

**Fig. 1:**
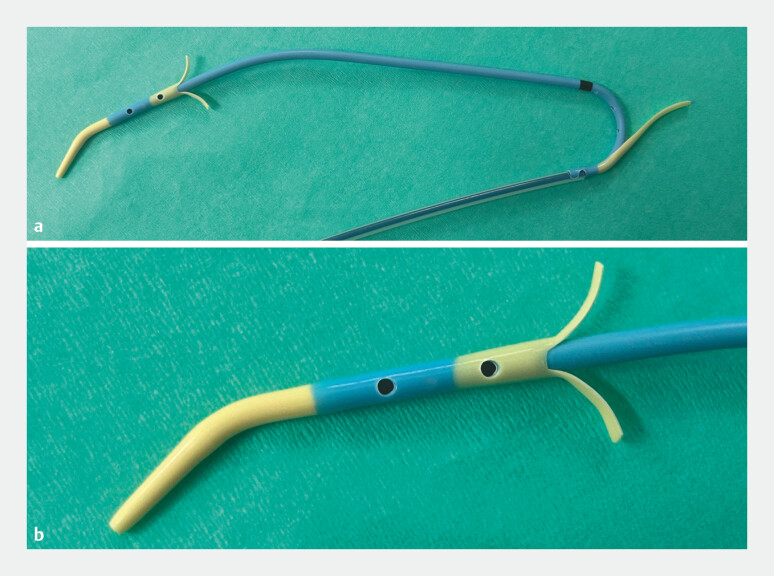
**a** The novel plastic stent (ADFlap, Century Medical, Tokyo, Japan) is equipped with double flaps to prevent migration. **b** The stent has a tapered tip, with a flap located at the distal 4 cm and two side holes, enabling bile drainage through both the tip and side holes.

In patients with perihilar cholangiocarcinoma (PHC) and obstructive jaundice undergoing planned major hepatectomy, preoperative biliary drainage of the future liver remnant is recommended, typically via endoscopic plastic stents (PSs 1 2 3 4 ). When endoscopic retrograde cholangiopancreatography (ERCP) is technically difficult, endoscopic ultrasound-guided hepaticogastrostomy (EUS-HGS) serves as a rescue procedure 5 ; however, PS placement via EUS-HGS typically drains only the left hepatic duct. We report a case of bilateral drainage achieved via EUS-HGS using a novel PS with a side hole at the tip ( Fig. 1 and Video 1 ).

EUS-guided hepaticogastrostomy with a novel plastic stent enabled preoperative bilateral drainage in patients with perihilar cholangiocarcinoma scheduled for central bisectionectomy.Video 1

**Fig. 2:**
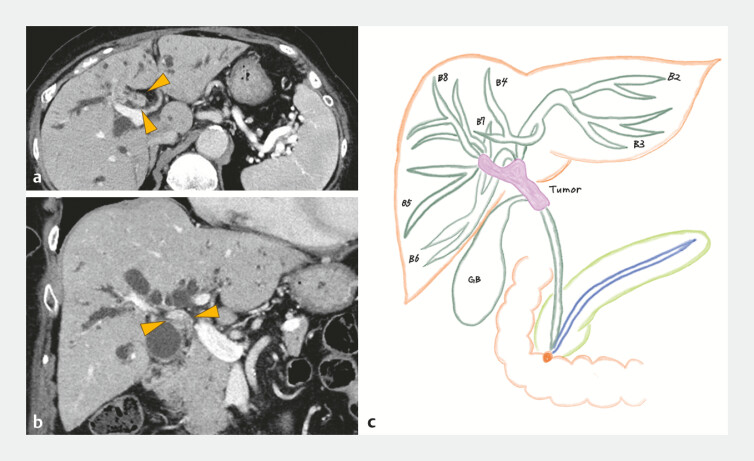
Computed tomographic (CT) images showed bile duct wall thickening and upstream biliary dilatation, leading to the diagnosis of bismuth type 3a perihilar cholangiocarcinoma. **a** Axial view. **b** Coronal view. **c** Schema based on CT findings.

**Fig. 3:**
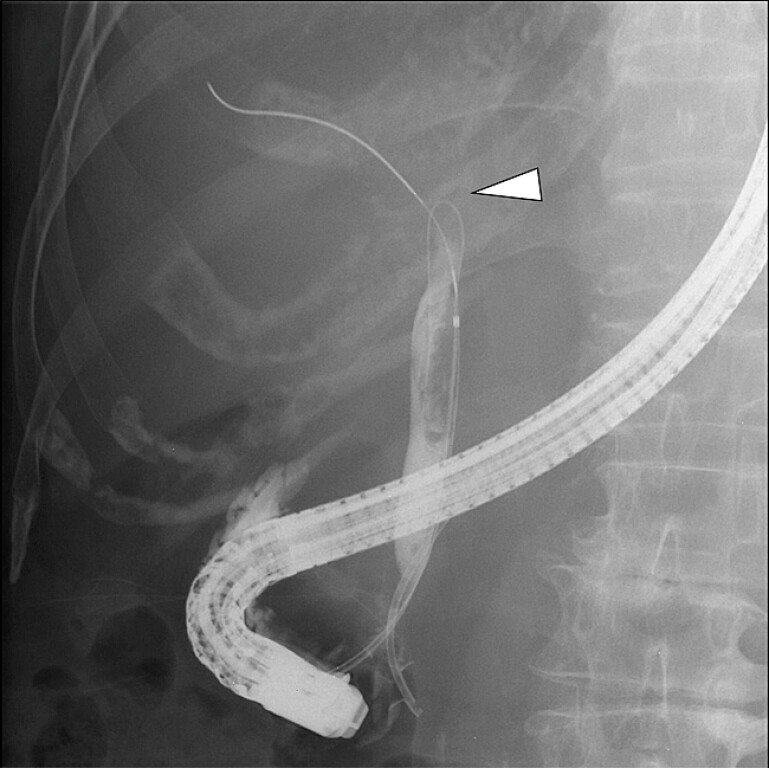
Cholangiography demonstrated a biliary stricture (arrowhead). While the guidewire cannulation of the anterior sectoral duct was successful, the posterior and left ducts could not be accessed.

An 84-year-old woman with jaundice was referred to our hospital. Contrast-enhanced computed tomography (CT) revealed bismuth type 3a PHC ( Fig. 2 ), and central bisectionectomy was recommended based on the remnant liver volume. ERCP was performed to drain the future liver remnant (the left and posterior sectoral ducts). Although the anterior sectoral duct was cannulated, the posterior and left sectoral ducts could not be accessed ( Fig. 3 ).

**Fig. 4:**
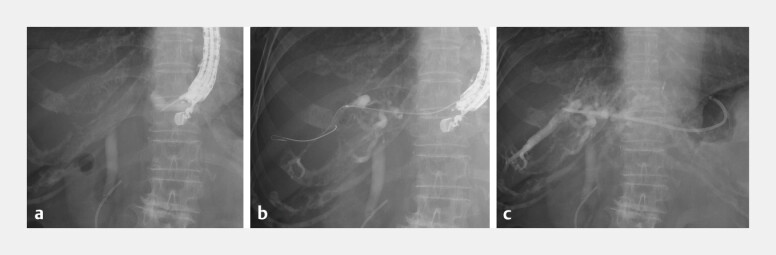
Endoscopic ultrasonography-guided hepatogastrostomy. **a** The dilated intrahepatic bile duct in segment 2 was punctured with a 19-gauge FNA needle. **b** A guidewire was inserted into the posterior bile duct. **c** The novel plastic stent was deployed with its tip positioned in the posterior duct and the side holes aligned with the left hepatic duct.

EUS-guided rendezvous was then attempted via puncture of the segment 2 bile duct using a 19-gauge FNA needle; however, guidewire advancement into the common bile duct failed. Nevertheless, the posterior sectoral duct was reached. To drain both ducts, a 7 Fr, 14-cm ADFlap stent (Century Medical, Tokyo, Japan) was deployed, with its tip in the posterior sectoral duct and the side hole aligned with the left hepatic duct, enabling effective bilateral drainage ( Fig. 4 ).

**Fig. 5:**
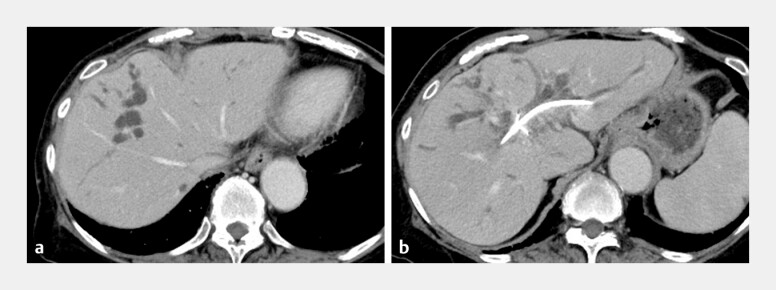
Follow-up computed tomography after EUS-guided hepaticogastrostomy. **a** Dilatation of both the posterior and left hepatic ducts has improved. **b** The tip of the stent is positioned within the posterior hepatic duct.

The patient experienced no postoperative complications. Jaundice improved, and follow-up CT confirmed decompression of both ducts ( Fig. 5 ). Although surgical resection was initially considered, conservative management was chosen by the patient. No further drainage was required during the 5-month follow-up period.

This case highlights the potential of a novel PS design in EUS-HGS to achieve bilateral drainage in complex PHC. Although feasible, this approach should be considered carefully, and its clinical role requires further evaluation in future studies.

Endoscopy_UCTN_Code_TTT_1AS_2AH
